# DO PREFERENCES FOR TELEWORK IMPACT JOB OPPORTUNITIES FOR OLDER JOB APPLICANTS? EVIDENCE FROM A HIRING EXPERIMENT

**DOI:** 10.1093/geroni/igae098.2374

**Published:** 2024-12-31

**Authors:** Samantha Brady, Lauren Cerino, Lisa D’Ambrosio, Joseph Coughlin

**Affiliations:** MIT AgeLab, Cambridge, Massachusetts, United States; Massachusetts Institute of Technology, Cambridge, Massachusetts, United States; MIT AgeLab, Cambridge, Massachusetts, United States; MIT AgeLab, Cambridge, Massachusetts, United States

## Abstract

Older adults represent an increasing share of the labor force, with adults aged 55 and older comprising almost one-quarter (24%) of the labor force in 2023 (U.S. Bureau of Labor Statistics, 2023). As the labor force ages, increasingly popular flexible work arrangements, like telework, can be especially beneficial at supporting older adults in the workforce. Although the labor force is aging and work is becoming increasingly conducive to prolonged working lives, age stereotypes and age discrimination remain rampant in today’s labor market, often limiting employment opportunities for older job seekers. And while telework provides benefits to older workers, requesting or utilizing telework arrangements may further exacerbate existing ageist stereotypes, as research prior to the pandemic found that teleworkers may be perceived as less competent and less committed employees. Using data from an original forced-choice conjoint experiment of hiring behaviors, this research causally examines the extent to which preferences for telework may further limit access to professional opportunities for job applicants of different ages. Preliminary analysis of pilot data (N=483) suggests that while preferences for certain telework arrangements negatively impact job opportunities for applicants of all ages, hiring penalties are especially large for the youngest and the oldest job applicants, compared to middle-aged applicants in our experiment. Analysis of complete sample data (N=2,000 hiring decision makers across the U.S. collected in March 2024) will also examine how telework preferences intersect with other job applicant attributes, including gender and caregiving responsibilities, to impact employment opportunities for particular groups of older job seekers.

